# Instruments to assess the role of the clinical pharmacist: a systematic review

**DOI:** 10.1186/s13643-022-02031-1

**Published:** 2022-08-22

**Authors:** Marina Oliveira Chagas, Tácio de Mendonça Lima, Flávio Rebustini, Matias Noll, Débora Penélope de Carvalho Queiroz, Janete Capel Hernandes, Neuma Chaveiro, Maria Alves Barbosa, Celmo Celeno Porto

**Affiliations:** 1 Department of Postgraduate in Health Sciences, Medical School, Federal University of Goiás, Goiânia, Goiás, Brazil; 2 Department of Pharmaceutical Sciences, Federal Rural University of Rio de Janeiro, Rio de Janeiro, Brazil; 3 School of Arts, Sciences and Humanities, University of São Paulo, São Paulo, SP Brazil; 4 Public Health, Federal Institute Goiano, Ceres, Goiás, Brazil; 5 Pharmaceutical Assistance Coordination, Jataí City Council, Jataí, Goiás, Brazil

**Keywords:** Clinical pharmacist, Pharmaceutical services, Professional performance instruments, Validity studies, Psychometric properties

## Abstract

**Background:**

The clinical pharmacist is an essential member of the healthcare team and plays an important role in health care in the primary care and the hospital setting. Knowledge regarding the instruments that evaluate the different activities of the clinical pharmacist, as well as the evaluation of the psychometric properties of these instruments, is necessary.

**Methods:**

A literature search was performed in the PubMed and Scopus electronic databases without time and language restrictions. For the search strategy, the “pharmaceutical services,” “validity studies,” and “professional performance” domains were used. To assess the quality of the instruments, the five sources of validity evidence of contemporary psychometry were used, and the Joanna Briggs Institute’s standardized instrument was used to assess the methodological quality of the studies. After screening 4096 articles, 32 studies were selected.

**Results:**

A total of 32 studies were included, and 32 instruments were identified to be used by pharmacists acting in various pharmaceutical practice scenarios. It was found that the available instruments were developed or adapted from others, with variation in the methods, constructs, dimensions, and domains, as well as the psychometric properties. Most of the instruments addressed community pharmacies, and evidence of content validity and internal structure was found most frequently. A standardized and validated instrument that comprehensively assessed the performance of the clinical pharmacist, addressing clinical activities, was not identified for all practice environments.

**Conclusions:**

Without standardized and validated instruments specifics to assess the performance of the clinical pharmacist, it is hard to establish the main clinical activities performed by pharmacists in their pharmaceutical practice environments and to propose training actions to improve professional practice. Despite the large number of instruments available and considered validated by the authors, it is questioned to what extent the validity indicators presented in the different studies really show the validation status.

**Systematic review registration:**

PROSPERO CRD 42018099912.

**Supplementary Information:**

The online version contains supplementary material available at 10.1186/s13643-022-02031-1.

## Background

The role of the pharmacist has evolved in recent decades. There is a change in the drug supply profile, moving towards patient-centered care in order to solve pharmacotherapeutic problems and improve the quality of life of patients. Thus, the clinical pharmacist must perform activities aimed at promoting the rational use of drugs, besides identifying, solving, and preventing potential and real problems related to pharmacotherapy and other health technologies having their practice redefined to carry out actions that meet the needs of people, family, caregivers, and the community [1].

Studies have demonstrated the expansion of this clinical role worldwide. In some countries such as Australia [2], the USA [3], and Germany [4], pharmacies are places where individuals obtain counseling on the management of the disease, review of the use of medication, prescription interventions, smoking cessation services, screening in the management of chronic diseases, and treatment of minor diseases. With this expanding practice scope, pharmacists are being recognized as key components in individualized patient care and as part of health teams [5]. In this sense, many researchers have developed instruments with the aim of evaluating this new role.

Evaluation instruments are useful and capable of presenting scientifically satisfactory results only when show robust evidence of validity. Despite the number of evaluation scales and instruments being developed, many are not validated and reproducible [6]. Validity was defined as “the degree to which evidence and theory support interpretations of test scores linked to the proposed uses of the tests” [7]. The evidence required for validity depends critically on the proposed interpretation of the scores and the properties of the test scores [8].

Considering this fact, this review aimed to identify and analyze the instruments that evaluate the role of the clinical pharmacist in different scenarios of pharmaceutical practice, as well as to assess the evidence of validity and the quality of the methodological procedures adopted.

## Methods

This review was registered at the International Prospective Register of Systematic Reviews (PROSPERO) [9] under the protocol number CRD 42,018,099,912. We report this review in accordance with Preferred Reporting Items for Systematic reviews and Meta-Analyses (PRISMA) guidelines [10] (Additional file 1).

### Search strategy and eligibility criteria

The articles were searched by two independent researchers in February 2020. The searches were performed using the PubMed and Scopus databases, without time and language restrictions. Two reviewers (MOC and DPCQ) independently performed the selection of articles through the databases. The search strategy consisted of a combination of different terms and keywords from the following three domains: (1) professional, (2) instruments, and (3) professional practice. The complete search strategy is presented in Additional file 2.

The inclusion of the articles considered studies that used instruments to evaluate the performance of the clinical pharmacist, and they were simultaneously as follows: (1) studies performed in different pharmaceutical practice environments, such as community pharmacies, hospital pharmacies, and outpatient pharmacies, (2) studies that evaluated the clinical activity of the pharmacist, and (3) studies with measures of validity and reliability. We excluded studies that (1) assessed other pharmaceutical activities, such as management, (2) evaluated patient satisfaction, (3) assessed the perception of other health professionals, and (4) used non-validated instruments.

### Study selection and data extraction

The selection of studies was based on the results of the research in the Mendeley Reference Manager software, with the removal of duplicates. Two reviewers (MOC and DPCQ) independently performed the selection of articles by reading the titles and abstracts. The selected texts were read in their entirety based on the established inclusion and exclusion criteria, and those articles that met the criteria were included in the study. Discrepancies between reviewers were carefully analyzed and resolved by a third reviewer (MN), who is a senior researcher.

Extraction of data was performed independently by the same reviewers (MOC and DPCQ) utilizing an Excel sheet. The collected information was as follows: (1) authors, (2) country, (3) study design, (4) language, (5) objectives of the study, (6) scenario, (7) measurement instrument, (8) sample, (9) instrument construct, (10) dimensions and domains, (11) sections, and (12) items, validity, and reliability.

### Assessment procedures

#### Assessment of methodological quality

The methodological quality of the studies was evaluated using standardized tools for critical evaluation of the Meta-Analysis of Statistics Assessment and Review Instrument of the Joanna Briggs Institute (JBI-MAStARI) [11]. Included studies were appraised by two reviewers (MOC, DPCQ) using the JBI critical appraisal checklist for analytical cross-sectional studies. Discrepancies between reviewers were carefully analyzed and resolved by a third reviewer (MN). This checklist contains the following items: (1) the sample was appropriate to address the target population, (2) criteria for inclusion in the sample clearly defined, (3) adequate sample size, (4) study subjects and the setting described in detail, (5) analysis conducted with sufficient coverage of the identified sample, (6) outcomes measured in a valid way, (7) objective and standard criteria for measurement, and (8) appropriate statistical analysis. The critical evaluation checklists used in this study were intended for analytical cross-sectional studies (Additional file 3). The risk of bias was classified as high when the studies reached up to 49% of the “yes” score; moderate when they reached 50 to 69% of the “yes” score, and low when the studies reached more than 70% of “yes” score [12].

### Assessment of psychometric quality

The assessment of the psychometric quality of methodological studies was carried out using the script of contemporary field of the state-of-the-art of psychometry [7, 8, 13]. Included studies were appraised by two reviewers (MOC, DPCQ). Discrepancies between reviewers were carefully analyzed and resolved by a third reviewer (MN). The five sources of validity evidence based for instruments considered for analysis were as follows: (1) content, (2) response process, (3) internal structure, (4) relations to other variables, and (5) consequences. Thus, the quality of each instrument for each study was assessed separately. They were classified according to the proposed criteria of the Cochrane Back Review Group [14] as strong (consistent positive results from multiple studies with good methodological quality or one study with excellent methodological quality), moderate (consistent positive results from multiple studies with fair methodological quality or one study with good methodological quality), limited (positive results from a study with fair methodological quality), conflicting (conflicting results from individual studies), or unknown (results from studies with poor methodological quality with an unknown level of evidence).

## Results

**Fig. 1:**
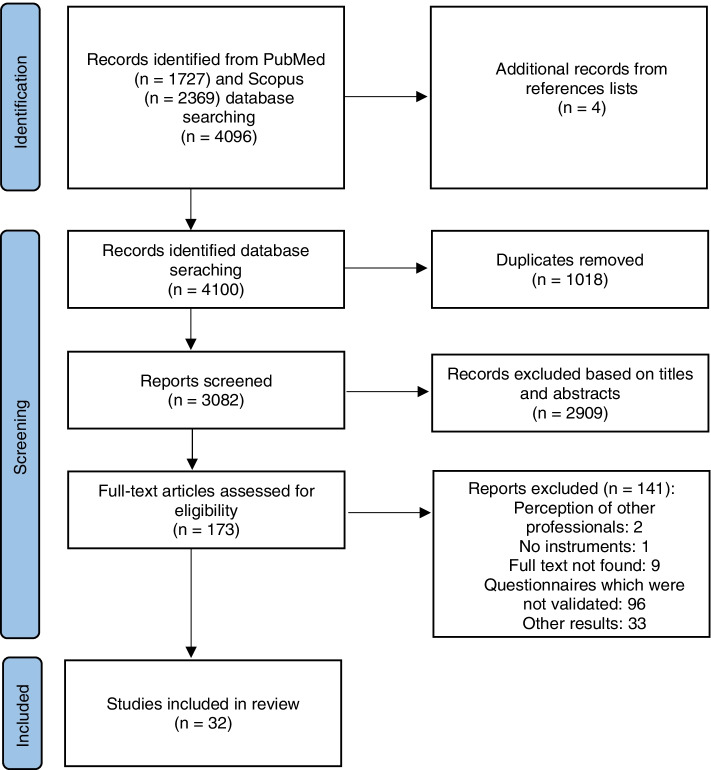
Flow diagram of study searching and selection process

The initial search results in 4096 records were identified in databases. Additional records were identified after reviewing reference lists with the inclusion of four studies. After removing 1018 duplicates, 3082 titles and abstract were reviewed, and 2909 articles were excluded. A total of 173 articles were included in full article review, and 32 publications were included in the final review (Fig. 1).

### Characteristics of the studies

The characteristics of the 32 studies are presented in Additional file 4. The year of publication ranged from 1994 to 2018 [15–46], and the study published in 1994 [35] was the first to be identified that used a questionnaire to investigate the involvement of community pharmacists in education and disease prevention activities. As for the language, 31 studies were published in English [15–46] and one in Spanish [45]. The sample size ranged from 69 to 4696 pharmacists [15–46].

Regarding the country of publication, the instruments were developed in North America (11 studies: 6 in Canada and 5 in the USA), Asia (11 studies: 3 in Qatar, 1 in Pakistan, 1 in Jordan, 1 in Malaysia, 1 in China, 1 in Thailand, 1 in Saudi Arabia, 1 in Indonesia, and 1 in Kuwait), Europe (7 studies: 2 in the UK countries, 1 in Denmark, 1 in Belgium, 1 in Spain, 1 in Serbia, and 1 comprising several European countries), Africa (2 studies in Sudan), and Oceania (1 study in Australian countries).

As for the study type, 22 were classified as methodological and cross-sectional, 5 methodological studies, and 5 cross-sectional studies. In methodological and cross-sectional studies, the creation of the instruments came from literature review, literature review and/or based on documents (standards, laws), and literature review and experts and authors’ consensus. Concerning the methodological studies that developed new instruments, the authors used qualitative (interviews/focus group) and quantitative approaches. In the five cross-sectional studies, the instruments came from adapted versions of other existing instruments.

### Characteristics of the instruments

The instruments are divided into “general” or “specific” according to the approached construct. Among the general instruments, ten instruments addressing pharmaceutical care [15, 17, 18, 20–24] were identified, and they evaluated the following: frequency in the identification and resolution of medication-related problems (assessment of the patient, monitoring of therapy, process documentation, and collaboration with other health professionals), attitudes in the practice of pharmaceutical care, and barriers to involvement in pharmaceutical care.

Regarding the specific instruments, they were divided into specific health/disease conditions (*n* = 9) and services/activities (*n* = 13). The instruments aimed at specific conditions evaluated the performance of the pharmacist for disease conditions [26–33] such as diabetes, depression, cardiovascular diseases, pregnancy/lactation, mental illness, smoking, and chronic diseases. In these instruments, the following issues were addressed: involvement in self-care services (counseling on therapies and diseases), dispensing activities, involvement in counseling in healthy living promotion, patient education, attitudes in providing services/activities, and the main barriers identified.

Regarding the specific instruments for services/activities [34–37, 39–45], the following were identified: instruments for education and health promotion, counseling in drug disposal, medication monitoring, reporting of adverse reactions, involvement in patient safety, acting on prescriptions, involvement in clinical research activities, drug therapy management service, pharmacotherapy follow-up service, immunization service, and oral health.

Regarding the location of practice, most of the studies were focused on pharmacists working in community pharmacies (*n* = 22). The others focused on hospital pharmacies (*n* = 4), different practice environments (*n* = 3), primary care pharmacies (*n* = 1), community and hospital pharmacies (*n* = 1), and community and outpatient pharmacies (*n* = 1).

In general, in relation to the structure, the instruments showed variations in the number of items (12 to 101 items). As for the sections, there was a variation between 2 and 6 sections, with 12 instruments not reporting the quantity, and regarding the domains and/or dimensions, only 13 instruments described and reported the quantities. Regarding validity, most studies presented information on content and internal structure.

The characteristics of the 32 instruments as well as their psychometric properties are described in Additional files 4 and 5, respectively.

### Quality of psychometric properties of instruments

The instruments were assessed for the five sources of validity evidence, which correspond to the content, the response process, the internal structure, other variables, and the consequences. In the analysis, not all presented the sources of evidence described, and the one that presented the best evidence was the BPCS instrument, as shown in Table 1.

**Table 1:** Levels of evidence of the quality of psychometric properties of the instruments

**Instruments**	**Author/year**	**Content**	**Response process**	**Internal structure**	**Other variables**	**Consequences**
**General Instruments**						
BPCS	Odedina and Segal (1996) [12]	+ + +	+	+ +	+ +	Na
Rossing’s questionnaire	Rossing et al. (2003) [16]	?	Na	+	Na	Na
Ngorsuraches’s questionnaire	Ngorsuraches and Li (2006) [17]	?	Na	?	Na	Na
Aburuz’s questionnaire	Aburuz et al. (2011) [19]	+	Na	+	Na	Na
Azhar’s questionnaire	Azhar et al. (2011) [20]	+	+	+	Na	Na
PABS	Jocić and Krajnović (2014) [21]	?	Na	-	Na	Na
Al-arifi’s questionnaire	Al-arifi et al. (2015) [22]	?	Na	?	Na	Na
El Hajj’s questionnaire	El Hajj et al. (2016) [23]	+	Na	?	Na	Na
**Specific instruments: disease/health conditions**						
Abduelkarem’s questionnaire	Abduelkarem et al. (2003) [25]	?	Na	+	Na	Na
Wibowo’s questionnaire	Wibowo et al. (2015) [26]	+	Na	?	Na	Na
El Hajj’s questionnaire	El Hajj et al. (2016) [27]	+	Na	+	Na	Na
Scheerder’s scale	Scheerder et al. (2008) [28]	+	Na	±	Na	Na
Albassam’s questionnaire	Albassam and Awad (2018) [29]	+	Na	+	Na	Na
Giannetti’s questionnaire	Giannetti et al. (2018) [30]	+	Na	?	Na	Na
Ashley’s questionnaire	Ashley (2007) [31]	?	Na	+ +	Na	Na
Mohamed’s questionnaire	Mohamed et al. (2014) [32]	+	Na	?	Na	Na
**Specific instruments: services/activities**						
Tai’s instrument	Tai et al. (2016) [33]	+	Na	+	Na	Na
Paluck’s questionnaire	Paluck et al. (1994) [34]	+ +	Na	+	Na	Na
Mohamed’s questionnaire	Mohamed et al. (2013) [35]	+	Na	+	Na	Na
Shah’s questionnaire	Shah and Chawla (2011) [36]	+	Na	?	Na	Na
MMAM instrument	Witry et al. (2016) [38]	+ +	Na	+ +	+	Na
Perreault’s questionnaire	Perreault et al. (2012) [39]	?	Na	+	Na	Na
Stewart’s questionnaire	Stewart et al. (2015) [40]	+	Na	+	Na	Na
Guirguis’s questionnaire	Guirguis et al. (2018) [41]	Na	Na	?	Na	Na
35 Elkalmi’s questionnaire	Elkalmi et al. (2014) [42]	+	Na	+	Na	Na
Taing’s questionnaire	Taing et al. (2016) [43]	+	Na	+	Na	Na
Zardain’s questionnaire	Zardain Tamargo et al. (2006) [44]	?	+	+ +	Na	Na

+  +  + or –- strong evidence positive/negative result, +  + or – moderate evidence positive/negative result, + or—limited evidence positive/negative result, + / − conflicting evidence, ? unknown, due to poor methodological quality, na no information available

### Methodological quality of the included studies

The studies were evaluated for methodological quality, as presented in Table 2. In the eight evaluated quality domains, eight studies met five or more quality criteria, and the others (*n* = 19) had less than five criteria assessed. The most common failed quality criterion by the studies was not having well-defined inclusion and exclusion criteria and not assessing confounding factors.

**Table 2:** Methodological quality of the included studies assessed by the Joanna Briggs Institute checklist for prevalence studies

**Instruments**	**Author/year**	Q1*	Q2*	Q3*	Q4*	Q5*	Q6*	Q7*	Q8*	**Score/risk**
**General instruments**										
BPCS adapted	Bell et al. (1998) [15]	N	Y	Y	Y	N	N	Y	Y	62.5%/moderate
Rossing’s questionnaire	Rossing et al. (2003) [16]	NC	Y	NC	Y	N	N	NC	Y	37.5%/high
Ngorsuraches’ questionnaire	Ngorsuraches and Li (2006) [17]	NC	Y	NC	Y	N	N	NC	Y	37.5%/high
BPCS adapted	Hughes et al. (2010) [18]	NC	Y	Y	Y	Y	N	Y	Y	75%/low
Aburuz’s questionnaire	Aburuz et al. (2011) [19]	N	Y	Y	Y	N	N	NC	N	37.5%/high
Azhar’s questionnaire	Azhar et al. (2011) [20]	NC	Y	Y	Y	N	N	NC	Y	50%/moderate
Al-Arifi’s questionnaire	Al-Arifi et al. (2015) [22]	NC	Y	Y	Y	N	N	NC	NC	37.5%/high
El Hajj’s questionnaire	El Hajj et al. (2016) [23]	Y	Y	NC	Y	N	N	NC	Y	50%/moderate
**Specific instruments: disease/health conditions**										
DAS adapted	Schapansky and Johnson (2000) [24]	N	Y	NC	Y	N	N	NC	Y	37.5%/high
Abduelkarem’s questionnaire	Abduelkarem et al. (2003) [25]	N	Y	Y	Y	N	N	N	NC	37.5%/high
Wibowo’s questionnaire	Wibowo et al. (2015) [26]	Y	Y	NC	Y	N	N	NC	Y	50%/moderate
El Hajj’s questionnaire	El Hajj et al. (2016) [27]	Y	Y	NC	Y	N	Y	NC	Y	62.5%/moderate
Scheerder’s scale	Scheerder et al. (2008) [28]	Y	Y	N	Y	N	N	N	Y	50%/moderate
Albassam’s questionnaire	Albassam and Awad (2018) [29]	NC	Y	NC	Y	N	Y	NC	NC	37.5%/high
Giannetti’s questionnaire	Giannetti et al. (2018) [30]	NC	Y	Y	Y	N	N	NC	N	37.5%/high
Ashley’s questionnaire	Ashley (2007) [31]	N	Y	Y	Y	N	N	Y	Y	62.5%/moderate
Mohamed’s questionnaire	Mohamed et al. (2014) [32]	Y	Y	NC	Y	N	N	NC	N	37.5%/high
**Specific instruments: services/activities**										
Tai’s instrument	Tai et al. (2016) [33]	N	Y	Y	Y	N	Y	NC	Y	62.5%/moderate
Paluck’s questionnaire	Paluck et al. (1994) [34]	NC	Y	Y	Y	N	N	Y	Y	62.5%/moderate
Mohamed’s questionnaire	Mohamed et al. (2013) [35]	Y	Y	NC	Y	N	N	NC	NC	37.5%/high
Shah’s questionnaire	Shah and Chawla (2011) [36]	NC	Y	NC	Y	N	N	NC	Y	37.5%/high
Isenor’s questionnaire	Isenor et al. (2018) [37]	Y	Y	NC	Y	N	N	NC	N	37.5%/high
Perreault’s questionnaire	Perreault et al. (2012) [39]	N	Y	NC	Y	N	Y	NC	NC	37.5%/high
Stewart’s questionnaire	Stewart et al. (2015) [40]	N	Y	Y	Y	N	N	Y	Y	62.5%/moderate
Elkalmi’s questionnaire	Elkalmi et al. (2014) [42]	N	Y	NC	Y	N	N	NC	Y	37.5%/high
Taing’s questionnaire	Taing et al. (2016) [43]	N	Y	NC	Y	N	N	NC	Y	37.5%/high
PSOPSC adapted	Jia et al. (2014) [46]	Y	Y	Y	Y	N	N	Y	Y	75%/low

*Y* Yes, *N* No, *NC* Not clear.*Q1, the sample was appropriate to address the target population. *Q2, criteria for inclusion in the sample clearly defined. *Q3, adequate sample size. *Q4, study subjects and the setting described in detail, *Q5, analysis conducted with sufficient coverage of the identified sample. *Q6, outcomes measured in a valid way. *Q7, objective and standard criteria for measurement. *Q8, appropriate statistical analysis

## Discussion

No other systematic review seeking to identify instruments to evaluate the performance of the clinical pharmacist in different pharmaceutical practice environments was found during this study. Thirty-two studies were included, and 32 instruments were identified to be used by pharmacists acting in various pharmaceutical practice scenarios. The instruments varied in terms of type (general and specific, according to the approached constructs), methods used (from literature review to mixed methods), and items, dimensions and domains, and psychometric properties (validity and reliability).

To our knowledge, there has been no instrument so far that assesses the role of clinical pharmacists in different environments of pharmaceutical practice. In this sense, there are some opportunities for future research, with the development of instruments that comprehensively assess the role of clinical pharmacists. Moreover, it can be applicable to all practice scenarios, and that it has standardized scales to compare studies.

A different approach was taken in this review compared to that in other studies, in which all instruments, regardless of the practice scenario, were analyzed. In another study, in which the objective was to evaluate instruments in clinical pharmaceutical practice, the authors Alshakrah, Steinke, and Lewis [47] limited the practice environment by selecting the instruments for the hospital environment. However, considering that clinical pharmacy covers not only hospital environments, our study sought to identify the tools for other environments, such as community and outpatient pharmacies.

In addition, the diversity of approaches to measuring pharmaceutical practice was broad for most analyzed criteria, particularly concerning the constructs and practice scenarios of interest for each study. This may be related to the different levels of knowledge and experience of pharmacists in each region of the country. This depends on the obtained training, additional qualifications in clinical pharmacy, and their individual fields of interest. For example, some pharmacists are active in the field of acute illness treatment, whereas others prefer to focus on chronic diseases, in addition to the different types of clinical services offered [48].

Regarding the construct, in the included studies, some instruments were aimed at evaluating the pharmaceutical performance in specific chronic diseases. Considering these instruments, studies that evaluated the performance of the pharmacist in the management of diabetes [25–27], cardiovascular diseases [28], and mental illnesses [29, 31] prevailed. In some cases, the country’s legislation emphasizes the necessity of involving pharmacists in the follow-up of chronic diseases [33].

Owing to this extensive approach, many of the instruments included were developed by the authors themselves as a secondary objective of the study. Therefore, 27 of them were classified as methodological and cross-sectional. In the analysis of the method, it was found that some authors did not describe the method, and others classified the studies only as cross-sectional. Considering that some of these studies developed instruments and followed criteria of validity and reliability, these were classified as methodological and cross-sectional.

For the development of these instruments, different methodologies were used, and the literature review with the adaptation of items from other studies was the most used type for the creation of the instruments’ items. Besides this technique, the literature review, combined with expert’s consensus techniques that were used for the development of some instruments in this study, was also reported in another review [49].

Some studies adapted validated instruments available in the literature, including the behavioral pharmaceutical care scale (BPCS), Pharmacy Survey on Patient Safety Culture (PSOPSC), and Diabetes Attitude Scale (DAS). The BPCS instrument [15] was the most extensively examined regarding the psychometric properties and the one offering more robust statistical results, being used and mentioned in many other studies. However, this instrument is to be applied only in the community pharmaceutical practice environment, which limits its use. The advantages of utilizing validated instruments include psychometric measures like validity and reliability, allowing the comparison of different studies. However, the limitations of the use of these instruments refer to the difficulties in finding an instrument for local use and meeting the proposed objectives. Furthermore, the adapted instruments did not follow the cross-cultural adaptation methodology.

Regarding the structure and the instruments dimension, the sample size in psychometric studies is based on the number of items in the instrument and aims to provide greater assurance in their analysis and quality [50, 51]. A sample of ten participants per item is considered acceptable, but there are studies that prove that twenty or more can significantly reduce error and inaccuracies in the solution of psychometric models, such as percentage of samples with correct factorial structure, average number of items classified incorrectly in the wrong factor, average error in eigenvalues, average error in factorial loads, the percentage of analyses that does not converge after 250 interactions, and percentage with Heywood cases [52].

The limitation in the sample requires that initial minimum parameters of adequacy, such as factorial loads, commonality, and the goodness-of-fit indexes, are greater than in studies with larger samples. In only two of the 32 articles analyzed, the ratio between the number of participants for each item of the instrument was greater than 20:1, and in five articles, the ratio was 10: 1. However, no study has reported whether the sample size was determined and whether this fact also guided the establishment of the minimum parameters model.

As for the reliability measure, most studies used Cronbach’s alpha with acceptable values within the established criteria. This coefficient depends on the magnitude of the correlation between the items and the number of items on the instrument [53]. Many studies have criticized the use of alpha without considering the nature and distribution of the data and the sample size, especially in samples involving more than 1000 participants [54, 55]. The use of McDonald’s Omega and greatest lower bound is preferable when there is data asymmetry, even in small samples and where high alpha values do not necessarily mean greater reliability and quality of the scales or tests, because they can be the result of long scales with parallel and redundant items or generate a restriction in the construct under study [56, 57].

Also, of the 32 studies analyzed, three performed test–retest reliability and did not validate the instrument’s construct. Bertchold 2016 [58] questions the use of the reliability term in the test–retest, reinforcing that Pearson’s correlation is a measure of association and not of reliability. Another way to clarify the reliability of an instrument and the possibility of guaranteeing its quality in different contexts is through invariance tests.

In this review, different instruments were identified to measure the performance of the clinical pharmacist, but several weaknesses were detected in the available instruments. According to the parameters and evidence criteria, few have undergone validation procedures with satisfactory results. Many authors refer to evidence from only one or two sources, such as reliability or correlation with the scores of another instrument, to support the validity of interpretations and, therefore, should be used with caution [59].

Concerning the methodological quality of the included cross-sectional studies, 55% of the studies had a high risk of bias. The JBI checklist addresses critical issues of internal and external validity that should be considered when assessing the validity of study prevalence data [60]. Therefore, the high risk of bias in these studies allows us to have more consistent and reliable conclusions from the data obtained, with important implications for data comparison.

Despite the large number of instruments available and considered validated by the authors, it is questioned to what extent the validity indicators presented in the different studies really show the validation status. We would like to highlight a potential limitation which need to be considered by the readers. The choice of two databases may have restricted the selection of more articles. In the selected databases, some studies may not have been found, as they are not indexed in the databases. On the other hand, as a strong point, we highlight that this is the first systematic review which has comprehensively synthesized existing evidence of performance of the clinical pharmacist in different pharmaceutical practice environments. Moreover, this dimensional approach allowed for a holistic view and a comparison between different instruments that assess clinical pharmacist performance. Furthermore, broad search strategies were run, which ensured a large number of studies were identified in field of clinical pharmacy.

## Conclusion

Thirty-two instruments which evaluated the role of the clinical pharmacist in different pharmaceutical practice scenarios, with weakness in the psychometric properties of the instruments and in the methodological quality of the studies. Also, a standardized and validated instrument that comprehensively assessed the performance of the clinical pharmacist, addressing clinical activities, was not identified for all practice environments. Thus, it is hard to establish the main clinical activities performed by pharmacists in their pharmaceutical practice environments and to propose training actions to improve professional practice.

## Supplementary Information

**Additional file 1.** Prisma checklist.

**Additional file 2. **Search strategy.

**Additional file 3. **JBI Critical Appraisal Checklist for Analytical Cross Sectional Studies.

**Additional file 4. **Characteristics of included studies.

**Additional file 5. **Psychometric properties of the included methodological studies.

## Data Availability

“All data generated or analyzed during this study are included in this published article [and its supplementary information files].”

## Abbreviations

BPCS: Behavioral pharmaceutical care scale
PSOPSC: Pharmacy Survey on Patient Safety Culture
DAS: Diabetes Attitude Scale
