# Supplementary material for: Instruments to assess the role of the clinical pharmacist: a systematic review
Source: Syst Rev. 2022 Aug 22;11:175. doi: 10.1186/s13643-022-02031-1 (PMC9396863; doi:10.1186/s13643-022-02031-1)
Supplement: Supplementary file 2 — Additional file 2. Search strategy. [file 13643_2022_2031_MOESM2_ESM.docx]

| **Bases de dados** | **Estratégias de busca** |
| --- | --- |
| PubMed) | (pharmacists[MeSH Terms]) OR clinical pharmacists[Text Word]) OR pharmaceutical services[MeSH Terms]) OR pharmaceutical care[Text Word]) OR community pharmacy services[MeSH Terms]) OR pharmacy services, hospital[MeSH Terms]) AND ((professional role[MeSH Terms]) OR attitude of health personnel[MeSH Terms]) AND (surveys and questionnaires[MeSH Terms]) OR evaluation studies[Publication Type]) OR validation studies[Publication Type]) |
| Scopus | (KEY ( pharmacists*OR*"clinical pharmacists"*OR*"pharmaceutical services" *OR*"pharmaceutical care" *OR*"community pharmacy" *OR*"hospital pharmacy" )  AND  KEY*(*"attitude of health personnel"*OR*"professional role" )   AND  KEY *(*surveys*OR*questionnaires*OR*evaluation*OR*validation) |

**Additional file 2. Search Strategy**
