# Supplementary material for: Instruments to assess the role of the clinical pharmacist: a systematic review
Source: Syst Rev. 2022 Aug 22;11:175. doi: 10.1186/s13643-022-02031-1 (PMC9396863; doi:10.1186/s13643-022-02031-1)
Supplement: Supplementary file 3 — Additional file 3. JBI Critical Appraisal Checklist for Analytical Cross Sectional Studies. [file 13643_2022_2031_MOESM3_ESM.doc]

**Additional file 3: JBI Critical Appraisal Checklist for Analytical Cross Sectional Studies**

Reviewer Date

Author Year Record Number

|  | Yes | No | Unclear | Not applicable |
| --- | --- | --- | --- | --- |
| 1. Were the criteria for inclusion in the sample clearly defined? | □ | □ | □ | □ |
| 1. Were the study subjects and the setting described in detail? | □ | □ | □ | □ |
| 1. Was the exposure measured in a valid and reliable way? | □ | □ | □ | □ |
| 1. Were objective, standard criteria used for measurement of the condition? | □ | □ | □ | □ |
| 1. Were confounding factors identified? | □ | □ | □ | □ |
| 1. Were strategies to deal with confounding factors stated? | □ | □ | □ | □ |
| 1. Were the outcomes measured in a valid and reliable way? | □ | □ | □ | □ |
| 1. Was appropriate statistical analysis used? | □ | □ | □ | □ |

Overall appraisal: Include □ Exclude □ Seek further info □

Comments (Including reason for exclusion)
