# Supplementary material for: Instruments to assess the role of the clinical pharmacist: a systematic review
Source: Syst Rev. 2022 Aug 22;11:175. doi: 10.1186/s13643-022-02031-1 (PMC9396863; doi:10.1186/s13643-022-02031-1)
Supplement: Supplementary file 4 — Additional file 4. Characteristics of included studies. [file 13643_2022_2031_MOESM4_ESM.docx]

**Additional file 4. Characteristics of included studies**

| Instrument | Authors (Year)/Country | Objectives | Study design | Target population/sample size | Results | Conclusion | Instrument availability |
| --- | --- | --- | --- | --- | --- | --- | --- |
| **General instruments** | | | | | | | |
|  | | | | | | | |
| BPCS | Odedina FT, Segal RJ (1996) / Florida (USA/America) | Develop and validate a behavioral pharmaceutical care scale (BPCS). | Develop a new instrument | Community pharmacists /  617 | Reliability coefficient for the domains was > 0.70 and the content validity index was 0.79. The nomological validity was established by confirming hypotheses in providing pharmaceutical care. | The instrument developed for measuring the efforts in pharmaceutical care supply was reliable, sensitive, and valid. | Available |
| BPCS modified version | Bell HM, et al. (1998) / Northern Ireland (United Kingdom/Europe) | Evaluate the extension of pharmaceutical care provided by community pharmacists in Northern Ireland. | Cross-sectional study | Community pharmacists /  230 | Higher score in the registration of patients' medication; good score in instrumental activities and low scores for reference and consultation activities. | Pharmacists routinely track prescriptions and maintain records of patients' medication and are less involved in the evaluation and documentation of patients' data. | Not available |
| Rossing’s questionnaire | Rossing C, Hansen EH, Krass I. (2003) / Denmark (Europe) | Investigate the provision of pharmaceutical care, in practice, in Denmark. In addition, determine the frequency in identifying medication-related problems (MRP) and the problem management process. | Cross-sectional study | Community Pharmacists /  218 | Most frequent activity: detect MRP; rarely or never set goals to solve and document the problems. | Pharmaceutical care, as defined in Denmark's policy documents, was not evident in practice. While some aspects of pharmaceutical care were performed, little documentation was reported | Not available |
| Ngorsuraches’ questionnaire | Ngorsuraches S, Li SC. (2006) / Thailand (Asia) | Examine the extent of pharmaceutical care practice and barriers experienced by Thai pharmacists in hospital and community pharmacies. | Cross-sectional study | Community pharmacy and hospital pharmacy / 314 | Pharmacists tended to identify and solve MRPs more than monitor drug therapy and rarely documented the processes. | Although pharmaceutical care is not incorporated into routine pharmacy practice, pharmacists had a good understanding and attitudes regarding care. The perceived barriers were lack of external cooperation, knowledge and skills, initiatives, and resources. | Not available |
| BPCS modified version | Hughe CM, et al. (2010) / Europe | Investigate the provision of pharmaceutical care by community pharmacists in Europe and study the various factors that could affect their implementation. | Cross-sectional study | Community pharmacy /  4696 (13 European countries) | Lower scores in the direct patient care dimension (particularly documentation, patient evaluation and implementation, therapeutic objectives, and monitoring plans) followed by performance evaluation and patient satisfaction assessment. Higher BPCS scores in Ireland, Germany, and Switzerland and higher overall level of pharmaceutical care provision. | The provision of pharmaceutical care in the community pharmacy is still limited in Europe. Pharmacists were routinely involved in general activities such as screening of medical records, but were rarely involved in patient-centered professional activities such as the implementation of therapeutic objectives and monitoring plans or in self-evaluation of performance. | Not available |
| Aburuz’s questionnaire | Aburuz S, et al. (2011) / Jordan (Middle East/Asia) | Answer the questions:  (a) Do community pharmacists understand pharmaceutical care?  (b) What is the extent of pharmaceutical care practice?  c) What are the main barriers to the practice of pharmaceutical care?  d) What is the attitude of community pharmacies when considering the provision of care for medicines? | Cross-sectional study | Community pharmacy /  291 | Over 62% of respondents had a correct understanding of the pharmaceutical care concept. The degree of pharmaceutical care provided was limited. In general, pharmacists have particularly good attitudes towards pharmaceutical care. Over 90% of respondents support the concept of pharmaceutical care. The need for training in pharmaceutical care was considered the main barrier to providing such care. | Although the provision of pharmaceutical care is limited at this stage in Jordan, the pharmacists who responded had a good understanding of pharmaceutical care. They expressed willingness to implement the pharmaceutical practice; however, they identified a few barriers to successful implementation. | Not available |
| Azhar’s questionnaire | Azhar S, Hassal, MA, Ibrahim, MMI. (2011) / Pakistan (Asia) | Investigate the perception of hospital pharmacists about their current clinical role in the health system in Pakistan. | Cross-sectional study | Hospital pharmacy / 116 | At least 42.2% of pharmacists were involved in drug education for patients. Although they were willing to take responsibility for solving drug-related problems, 84.5% of them stated that their current role is more focused on pharmacy record  Keeping; 57.8% indicated they were involved in compiling and updating hospital medication forms. | The findings suggest that pharmacists in Pakistan have concerns regarding the performance of their professional roles and face significant barriers in engaging with clinical services. However, they need to be proactive in their collaboration with other health professionals and in the concept of pharmaceutical care to establish themselves in health systems. | Not available |
| PABS | Jocić D, Krajnović D. (2014) / Serbia (Europe) | Describe the development and psychometric validation of a research tool to assess attitudes and behavior of pharmacists in relation to working with patients (Pharmacists Attitude and Behavior Scale, PABS). In addition, determine the reliability, validity, and factorial structure of a newly constructed instrument - PABS. | Develop a new instrument | Primary care community pharmacy /  123 | Cronbach's alpha coefficient was 0.67. Factorial analysis of the main components was performed and 7 factors with latent roots above 1 were extracted, explaining 64.92% of the total variance; a single factor explained 30.84%, 8.20%, 6.55%, 5.63%, 5.01%, 4.68%, and 4.01% of variance. Based on the factorial analysis results some items in the scale were excluded (totally 7), so the revised PABS form contained a total of 23 items. | The initial PABS scale did not meet the statistical criteria of reliability (Cronbach's alpha coefficient < 0.7); however, the findings indicated its potentially acceptable possibility of construct validity. The results support its use as a tool to evaluate the behavior of pharmacists in day-to-day practice and provide its use as an indicator of pharmaceutical care quality. | Available |
| Al-Arifi’s questionnaire | Al-Arifi MN, et al. (2015) / Saudi Arabia (Middle East/Asia) | Explore pharmacists’ opinions regarding pharmaceutical care (PC) in Riyadh, capital of Saudi Arabia. | Descriptive, cross-sectional study | Community pharmacists in primary care center / 80 | Pharmacists in general have expressed positive attitudes towards PC. A total of 73 (91%) interviewees indicated a common situation regarding prescription adequacy; they identified errors in prescriptions with incomplete information. Most respondents (78.6%) disagreed or totally disagreed with the statement that pharmacists in primary health centers do not have enough knowledge/skills to provide PC. | The findings of this study disclosed that pharmacists have positive attitudes towards PC in general. More attention should be given to improving the current capabilities to extend their professional roles. However, further development in Saudi hospitals would be recommended to improve health systems. | Not available |
| El Hajj’s questionnaire | El Hajj MS, Al-Saeed HS, Khaja M. (2016) / Qatar (Asia) | Examine the extent of pharmaceutical care practice and barriers to pharmaceutical care provision by Qatar's pharmacists and assess the level of understanding of pharmaceutical care and their attitudes towards PC provision. | Cross-sectional study | Community pharmacists, (networks/independent), hospital pharmacists (public and private), and pharmacists of outpatient clinics / 255 | Over 80% of respondents understood the purpose of PC and the role of the pharmacist in PC. Only 47% recognized the role of the patient in PC and 35% were aware of the differences between clinical pharmacy and PC. More than 80% of respondents had correct  understanding of the aim of PC and of the pharmacist role in  PC.The perceived barriers included inconvenient access to the patient's medical information (78%) and lack of personnel and time (77 and 74%, respectively). | Although PC is not incorporated into pharmacy practice, pharmacists have shown positive attitudes towards its provision. Future work should focus on improving the understanding of PC and overcoming all barriers. | Available |
| **Specific Instruments** | | | | | | | |
| **Disease / Health Status** | | | | | | | |
| DAS modified version | Schapansky LM, Johnson JA. (2000) / Canada (America) | Evaluate the attitudes of pharmacists towards diabetes; assess the measurement properties of the diabetes attitude scale (DAS) in a sample of pharmacists and estimate the number and attitudes of pharmacists certified as diabetes educators (CDE). | Cross-sectional study | Community, hospital and other pharmacists /  339 | The attitudes of pharmacists varied according to the practice environment, year of graduation, and whether they had diabetes or a CDE designation. Most factorial scores were reliable. | Pharmacists agree that they should be part of the healthcare team to manage diabetes, should have specialized training to provide primary care to the patient with diabetes, and that they have the skills to become diabetes educators. | Not available |
| Abduelkarem’s questionnaire | Abduelkarem AR, et al. (2003) / United Kingdom (Europe) | Describe the viewpoints and practices of community pharmacists regarding services for people with type 2 diabetes. | Cross-sectional study | Community pharmacists /  165 | Over 80% “frequently/very often” saw patients when they received their prescriptions; three quarters “never/very rarely” or “sometimes” advised patients about drugs and disease; over 90% believed in a healthy lifestyle, but most “never/very rarely” were involved in its promotion; 10% “frequently/very often” promoted regular eye exams; 50% promoted home glucose monitoring; most checked prescriptions for drug interactions. | This study found that community pharmacists' councils and services for people with type 2 diabetes fell short of the standards and objectives set by the National Diabetes Service Structure. These findings can be used to promote discussion within the profession and with interested parties about the future role of the community pharmacist in diabetes treatment. | Not available |
| Wibowo’s questionnaire | Wibowo Y, et al. (2015) / Indonesia (Asia) | Assess community pharmacy services and the roles of pharmacists in type 2 diabetes care and associated characteristics (pharmacist and pharmacy) with current practice. | Cross-sectional study | Community pharmacists /  240 | Drug dispensing (100%) and drug use education (72.6%) were the most common practices; the highest priorities of the services, beyond dispensing, were drug education [use instructions (58.6%) and common/important adverse effects (25.7%)], exercise education (36.5%), food education (47.7%), and monitoring drug adherence (27.9%). The main barrier identified was the low availability of pharmaceutics (for food education, as well as compliance monitoring). | Most community pharmacies only provided the basic dispensing service for patients with type 2 diabetes. Many pharmacists believed they should expand their roles particularly in relation to patient monitoring and education. Developing the professional role of the pharmacist would help manage the growing incidence of diabetes. | Available |
| El Hajj’s questionnaire | El Hajj MS, et al. (2016) / Qatar (Asia) | Evaluate the involvement of pharmacists in health promotion of cardiovascular diseases (CVD) in Qatar to identify the activities they currently provide to patients with CVD risk factors, describe their attitudes toward their involvement in CVD prevention, and assess the barriers to providing CVD prevention services. | Cross-sectional study | Community pharmacists, pharmacists of outpatient clinics, and pharmaceutics of outpatient hospitals /  141 | 70% responded “seldom/never” to 6 of the 10 health promotion activities of CVD. 84% and 68% “always/frequently” describe the time to use antihypertensive drugs and the adverse effects of the drugs, respectively. 50% “rarely/never” review drug history or offer adherence interventions. Lack of educational materials was the main barrier (55%), plus lack of private counseling area (44.6%), and lack of time (38.3%). Women and community pharmacists were more involved in promoting CVD health. | The scope of CVD prevention practice in pharmacy is limited in Qatar. Efforts need to be made to increase the involvement of pharmacists in the prevention of CVD. | Not available |
| Scheerder’s scale | Scheerde G, De Coste I, Van Audenhove C. (2008) / Belgium (Europe) | Assess attitudes, current practices, and barriers to treating people with depression. | Cross-sectional study | Community pharmacists /  69 | Most pharmacists have an incredibly positive attitude towards their role in treating depression. This attitude has been reflected in current practice, and fulfilling this role seemed to be more difficult with patients with depression than with patients with other physical conditions. Lack of training in mental health issues was the most important barrier reported as well as the low level of cooperation with professionals. | For pharmacists to effectively engage in treatment for depression, barriers need to be addressed through specific training programs and improved cooperation with professionals. | Not available |
| Albassam’s questionnaire | Albassam A, Awad A. (2018) / Kuwait (Middle East/Asia) | Identify the services provided by community pharmacists in Kuwait and their views on self-care during pregnancy and lactation. | Cross-sectional study | Community pharmacists /  192 | Main services were recommendations of vitamins and food supplements (89.8%) and advice on contraception (83.4%). More than half would recommend medications for headache, constipation, and cough, among others. In cases of diarrhea, hemorrhoids, insomnia, varicose veins, swelling of feet and legs, vaginal itching, and back pain, among others, patients were referred to the doctor. When offering advice and solving health problems they had enough knowledge (61.5% and 50.5%, respectively) and confidence (58,3% and 53,1%, respectively). The majority (88.5%) agreed on a continuing education program for their practice. | The results show that respondents offered different recommendations for the treatment of pregnancy and lactation-related diseases. They also highlight the need for interventions, including continuing professional development and reviewing the undergraduate pharmacy curriculum. | Available |
| Giannetti’s questionnaire | Giannetti V, et al. (2018) / USA (America) | Assess demographic characteristics, service provision characteristics, stigma, attitudes, and behaviors of community pharmacists with mental illness. | Cross-sectional study | Community pharmacists /  239 | For pharmacy services, ratings were higher for the disposition/interest than for comfort/confidence. Those who reported providing medication therapy management (MTM) services and with personal experience reported greater comfort, confidence, disposition, and interest. The increased provision of pharmaceutical services was significantly associated with willingness and interest in providing specific services for mental illness. | Despite the willingness/interest in providing services to patients with mental illness, reduced levels of comfort/confidence remain barriers related to services for community pharmacists. | Available |
| Ashley’s questionnaire | Ashley MJ, Victor JC, Brewster J. (2007) / Canada (America) | Investigate pharmacists' involvement in smoking cessation in four provinces. | Cross-sectional study | Community pharmacists /  962 | 70% had positive attitudes towards smoking cessation; 50% thought that pharmacists have an important role in motivating patients to quit and in most aspects of motivation, care, and referral. In all provinces, less than 40% intervened in various ways in the previous year, with more than half of their patients who smoked. Counseling to reduce or quit, trying to increase motivation to quit, and suggesting the use of nicotine replacement therapy were the most frequent interventions. | The results provide a baseline for provincial monitoring of pharmacists' attitudes toward smoking cessation, role perceptions, and interventions. They can also provide tobacco control initiatives. | Not available |
| Mohamed’s questionnaire | Mohamed SS, Mahmoud AA, Ali AA. (2014) / Khartoum (Sudan / Africa) | Describe the current and potential role of Sudanese community pharmacists in response to symptoms (RTS) and chronic disease management (CDM) and identify perceived barriers. | Cross-sectional study | Community pharmacists /  183 | Most respondents (90%) reported being involved in RTS activities, with negative opinions on practice standards. They do not have specific lists on the treatment of minor conditions (87.4%), counseling procedures (84.7%), and reference forms (85.8%). They see an important role in CDM (95.3%) and accept teamwork with other health care providers (87.5%). Lack of knowledge and training, time, space, acceptance of patients, and a new role for pharmacists were some of the barriers identified. | Need for training and clinical knowledge and well-defined practice standards were identified. The focus on product activities needs to be refined to include more patient-centered services. To improve self-care services for patients, several obstacles have been identified that need to be resolved. This requires collaboration between different parties, including academics, government agencies, and professional organizations. | Not available |
| **Services/Activities** | | | | | | | |
| Tai’s instrument | Tai B, et al. (2016) / USA (America) | Assess community pharmacists' knowledge of drug disposal and examine the usefulness of the theory of planned behavior (TPB) in predicting their intention to provide drug disposal education to their patients. | Descriptive and cross-sectional study | Community pharmacists /  142 | (n = 142). Demonstrated a positive intention to provide education but the majority (67.9%) offered this information once a month or less. Attitude, subjective norms, and perceived behavioral control were significant predictors of intention, representing 40.8% of the variation in the intention to provide education on disposal. The reliability scale ranged from 0.596 to 0.619 for the four constructs. Few pharmacists accurately selected all appropriate substance disposal recommendations for non-controlled and controlled substances. | Pharmacists demonstrated favorable attitude, subjective norms, perceived behavioral control, and intent to provide this education. However, their knowledge in this field may be insufficient and they may not be able to provide this information to their patients. | Not available |
| Paluck’s questionnaire | Paluck EC, et al. (1994) / British Columbia-Canada (America) | Determine to what extent community pharmacists in British Columbia participate in health education and disease prevention activities. | Cross-sectional study | Community pharmacists /  485 | Pharmacists participate more often in health education/disease prevention activities related to the dispensing or selling of medicines. Activities with less participation included talking to community groups about health-related problems; participating in disease screening programs; consulting clients about their level of occupational stress; advising clients on AIDS prevention; and consulting clients about their smoking status. | In conclusion, community pharmacists are involved in disease prevention activities to a limited extent. Pharmacists should take the initiative to provide health education/disease prevention services, as the client is often unaware of the pharmacist's capabilities in this field. | Not available |
| Mohamed’s questionnaire | Mohamed SS-E, Mahmoud AA, Ali AA. (2013) / Sudan (Africa) | Assess the attitude of community pharmacists in education and health promotion and screening and assess the extent of involvement in each activity and identify barriers to its provision. | Cross-sectional study | Community pharmacists /  183 | Most (>70%) are willing to participate in public health services and have consented to provide more effective education and health promotion services. A high proportion is providing health information on a variety of subjects such as healthy diet, obesity and weight reduction, exercise, smoking cessation, and contraception methods. However, this involvement is possible during the prescription analysis. Blood pressure and blood glucose screening are acceptable, and barriers are lack of time, training, and professional recognition. | The provision of these activities is favored post training and removal of barriers. | Not available |
| Shah’s questionnaire | Shah B, Chawla S. (2011) / New York (USA/America) | (1) Evaluate the current extent of MTM provision in New York; (2) investigate pharmacists' attitudes, efforts, interests, and challenges in providing MTM; and (3) examine factors that influence pharmaceutical interest in providing MTM. | Cross-sectional study | Community pharmacists /  93 | Pharmacists had slightly positive attitudes towards the provision of MTM and were extremely interested in providing some MTM services. There was a positive correlation between the pharmacist's attitude towards MTM and interest in providing it. Of the six listed challenges to MTM provision, respondents indicated further time spent with additional patients and pharmacists as the most difficult challenge to overcome. | This study is an evaluation of the needs of New York City pharmacists regarding the provision of MTM services. | Not available |
| Isenor’s questionnaire | Isenor JE, et al. (2018) / Canada (America) | Describe the experiences of pharmacists in the Canadian province of New Brunswick as immunizers, including administered vaccines and perceived barriers and as facilitators in providing immunizations. | Cross-sectional study | Community pharmacists /  168 | Over 75% reported administering hepatitis A and B, influenza, and zoster vaccines. Most felt they were accepted as immunization providers by patients, local doctors, and the provincial health department. Most reported barriers were lack of a universally funded influenza immunization program, insufficient space and personnel, and concerns about reimbursement for services. | Pharmacists in New Brunswick, Canada are actively participating in providing a variety of immunizations and have felt fully supported by patients and other healthcare professionals. The identified barriers may provide information for other jurisdictions, considering the expanding role of pharmacists as immunizers. | Not available |
| MMAM instrument | Witry MJ, et al. (2016) / USA (America) | The goals were: (1) to create a measure of the community pharmacist's drug monitoring attitude; (2) to test competing validity using a validated measure of drug monitoring behaviors; and (3) to report the community pharmacist's attitudes towards drug monitoring. | Develop a new instrument | Community pharmacists /  254 | Factorial analysis yielded two domains: an internal one with seven items and positive words (α = 0.819) and an external domain with eight items and negative words (α = 0.811). Both domains correlated positively with the monitoring domain of the BPCS for the convergent validity. Pharmacists were more oriented towards the side effect and effectiveness of drug monitoring than to nonadherence monitoring. | The process of item development by mixed methods has created a reliable and valid measure of a pharmacist's drug monitoring attitude. While pharmacists have had an overall positive attitude towards drug monitoring, improvements are needed to strengthen adherence to monitoring and to make pharmacy environments more favorable for monitoring. | Not available |
| Perreault’s questionnaire | Perreault MM, et al. (2012) / Canada (America) | Describe the experience of Canadian intensive care pharmacists' in clinical research, present their views about clinical research, and identify factors that facilitate their involvement in clinical research. | Cross-sectional study | Hospital pharmacists /  215 | Overall, 123 (58.6%) were minimally involved in the research; 97.2% believed that involvement in research was desirable and many expressed interest in being more involved in the research; 99.5% agreed that more support should be provided to pharmacists who are interested in research. | Canadian intensive care pharmacists are involved at different levels in clinical research and are extremely interested in initiating and supporting research activities. Opportunities are present, but there are significant barriers. The value of research initiated by the pharmacist needs recognition as a priority within hospital pharmacy management. | Not available |
| Stewart’s questionnaire | Stewart D, et al. (2015) / Qatar (Asia) | Determine interests, experience, and trust of hospital pharmacists employed by Hamad  Medical Corporation (HMC) in Qatar regarding research and attitudes towards research and facilitators and barriers. | Cross-sectional study | Hospital pharmacists /  213 | Four components were identified: general attitudes toward research; confidence, motivation, and resources; research culture; and support. Although they were generally positive for all items, they were less sure of resources to conduct research, access to training, and statistical support. Half of the respondents never thought about getting involved in research. | HMC pharmacists expressed high levels of interest in research in comparison to experience and confidence. While general attitudes towards research were positive, there were some barriers related to support (e.g., administration) and research culture. The findings are of fundamental relevance when considering the objectives of encouraging research, improving skills, and identifying skill gaps. | Available |
| Guirguis’s questionnaire | Guirguis LM, et al. (2018) / Canada (America) | Develop a questionnaire to assess the factors influencing the adoption of prescription by pharmacists (i.e., continue, adapt, or initiate therapy), describe the use of pre-incentive and mixed mode research, and establish the psychometric properties of the research. | Develop a new instrument | Different practice environments /  378 | The factor analysis led to 27 questions on eight scales: (1) self-efficacy, (2) practice environment support, (3) interprofessional relationship support, (4) impact on professionalism, (5) impact on patient care, (6) prescription behaviors, (7) technical use of the electronic health record (EHR), and (8) use of EHR in patient care. Prescription beliefs and technical use of EHR scales showed low reliability, whereas the remaining six scales displayed strong evidence of reliability and validity. | Through a multi-step process, a research tool has been developed to capture the perceptions of pharmacists about prescriptive influences. This questionnaire may support future research in developing interventions to improve the adoption of prescriptions and enhance direct patient care by pharmacists. | Not available |
| Elkalmi’s questionnaire | Elkalmi RM, et al. (2014) / Malaysia (Asia) | To explore consciousness and attitudes of community pharmacists towards the national adverse drug reaction (ADR) notification system in the states of Northern Malaysia. | Cross-sectional study | Community pharmacists /  104 | 72.1% were unaware of the pharmacovigilance activities performed by the Malaysian drug regulatory authority; 61.5% emphasized the importance of ADR reports, and only 12.9% sent ADR reports to the Advisory Committee (MADRAC). Barriers included lack of reporting skills (34.7%), unavailability of reporting forms (42.6%), and ignorance regarding where the report should be sent (44.6%). | Findings revealed that pharmacists have a positive attitude towards the ADR reporting system in the country. However, these findings highlight the urgent need for special education programs to establish efforts to promote ADR reporting. | Not available |
| Taing’s questionnaire | Taing M, et al. (2016) / Australia (Oceania) | Investigate attitudes, behaviors, and practices of the community pharmacist for oral health in the Australian scenario, describe the frequency and nature of oral health consultations and obtain information on smoking cessation support for people with oral health problems. | Cross-sectional study | Community pharmacists /  144 | 93% believed that providing oral health advice was their role; 97% believed that additional education would benefit their practice. A small proportion (8%) always asked about patients' smoking habits and almost all pharmacists (97%) wanted additional education and training to benefit their oral health practice. | Australian pharmacists have an important role in oral health and there is an opportunity to enhance this role and address risk factors. The conclusions of this study may guide future research on the development of training programs, standards, and improved oral health care practices for Australian pharmacists. | Available |
| Zardain’s questionnaire | Zardain TE, et al. (2006) / Spain (Europe) | Validate a questionnaire to measure the attitude, social influence, and self-efficacy (ASE) psychosocial determinants of pharmacotherapeutic driven follow-up (STF) and the stage of change according to Prochaska and DiClemente, in pharmacy office pharmacists. | Develop a new instrument | Community pharmacists /  134 | Stability was guaranteed in the 85 respondents to the test and retest process. From the exploratory factor analysis (construct validity), 4 factors and 104 items were obtained. These factors showed high reliability (Cronbach's reliability between 0.87 and 0.96). Discriminant validity: comparing groups in extreme stages of change, there were significant differences in attitude, motivation, and self-efficacy. | The repeatability was high, and the questionnaire seems valid and reliable enough. | Not available |
| PSOPSC modified version | Jia PL, et al. (2014) / China (Asia) | Explore patient safety culture attitudes and perceptions among pharmacy workers in China using a pharmacy survey on patient safety culture (PSOPSC) and assess the psychometric properties of the Chinese translated version of the PSOPSC. | Cross-sectional study | Hospital pharmacists /  527 | The rate of positive response in three dimensions (“Teamwork,” “Team training and skills”, and “Team, pressure at work and pace”) was higher than that of the Agency for Health Research and Quality (AHRQ). There was a statistical difference in the perception of patient safety culture in different hospitals and levels of qualification. The internal consistency of the total was comparatively satisfied (Cronbach's α 0.89). There was a statistical difference in the perception of patient safety culture in different hospitals and levels of qualification. The internal consistency of the total was comparatively satisfied (Cronbach's α 0.89). | The results showed that among the pharmacies surveyed in China, there was a positive attitude towards patient safety culture organizations. Patient safety perspectives were identified at different hospitals and at healthy qualification levels that can help support decisions about actions to improve safety culture in pharmacy configurations. The Chinese translation of the PSOPSC applied in this study is acceptable. | Available |
