# Supplementary material for: Instruments to assess the role of the clinical pharmacist: a systematic review
Source: Syst Rev. 2022 Aug 22;11:175. doi: 10.1186/s13643-022-02031-1 (PMC9396863; doi:10.1186/s13643-022-02031-1)
Supplement: Supplementary file 5 — Additional file 5. Psychometric properties of the included methodological studies. [file 13643_2022_2031_MOESM5_ESM.docx]

**Additional file 5**. **Psychometric properties of the included methodological studies**

| **Instrumentos** | **Autor/ Ano / País** | **Latent variable** | **Content** | **Response Processe** | **Internal Structure** | **Relations others variables** | **Consequences** |
| --- | --- | --- | --- | --- | --- | --- | --- |
|  |  |  |  |  |  |  |  |
| BPCS | Odedina FT. et al (1996) / Flórida [15] | Behavioral Pharmaceutical care | Developed from a literature review and focus group; Content Validity: experts and pretest | NR | Internal consistency: Cronbach’s alpha> 0.70  Confirmatory Factor Analysis of the 14 domains | Convergent and divergent validity | NR |
| Rossing’s questionnaire | Rossing, C. e al (2003) / Dinamarca [17] | Pharmaceutical care | NR the creation procedures item;  External validity | NR | Internal consistency: Cronbach’s alpha 0.89 | NR | NR |
| Ngorsuraches’s questionnaire | Ngorsuraches, S. et al (2006) / Tailândia [18] | Pharmaceutical care | Developed from a literature review; External validity for non-respondents | NR | Internal consistency: Cronbach’s alpha for attitude section = 0.7 | NR | NR |
| Aburuz’s questionnaire | Aburuz S. et al (2011) / Jordânia [20] | Pharmaceutical care | Developed from the literature review and guidelines; Content validity | NR | Internal consistency: Cronbach’s alpha > 0,8 | NR | NR |
| Azhar’s questionnaire | Azhar, S. et al (2011) / Paquistão [21] | Pharmaceutical care | Content validity: four professionals and two focus groups | NR | Internal consistency: Cronbach’s alpha = 0,76 | NR | NR |
| PABS | Jocić, D. et al (2014) / Sérvia [22] | Attitudes and  beliefs of pharmacists toward their work with patients | Developed from the literature review; Pre-tested with 7 pharmacists | NR | Internal consistency: Cronbach’s alpha = 0.67; PCA and varimax rotation for 7 factors | NR | NR |
| Al-arifi’s questionnaire | Al-arifi, M.N. et al (2015) / Arábia Saudita [23] | Pharmaceutical care | Developed by adapting other studies; Face validity | NR | Internal consistency: Cronbach’s alpha = 0,66 | NR | NR |
| El Hajj’s questionnaire | El Hajj, M. S. et al (2016) / Catar [24] | Pharmaceutical care | Developed from a literature review.  Content validity: 4 members and pre-test | NR | Internal consistency: Cronbach’s alpha = 0,93 | NR | NR |
|  |  |  |  |  |  |  |  |
|  |  |  |  |  |  |  |  |
| Abduelkarem’s questionnaire | Abduelkarem, A. R. et al (2003) / Reino Unido [26] | Type 2 diabetes | Developed from guidelines; Face validity | NR | Internal consistency: Cronbach’s alpha = 0.79; Test-retest = 0.73 | NR | NR |
| Wibowo’s questionnaire | Wibowo, Y. et al (2015)/ Indonésia [27] | Diabetes | Developed from the literature;  Content validity and Face validity: panel of seven academics | NR | Test-retest using Kappa = 0.4 to 1 | NR | NR |
| El Hajj’s questionnaire | El Hajj, M. S. et al (2016) / Catar [28] | Cardiovascular diseases | Developed from a literature review; Content validity: 4 members and pretest | NR | Internal consistency: Cronbach’s alpha = 0,93 | NR | NR |
| Scheerder’s scale | Scheerder, G. et al (2008) / Bélgica [29] | Depression | Content and Face validity | NR | Internal consistency: Cronbach’s alpha = 0,76 | NR | NR |
| Albassam’s questionnaire | Albassam, A. et al (2018) / Kuwait [30] | Pregnancy and breastfeeding | Content and Face validity | NR | Internal consistency: Cronbach’s alpha > 0,7 for all sections | NR | NR |
| Giannetti’s questionnaire | Giannetti, V. et al (2018) / EUA [31] | Mental llness | Face validity | NR | Internal consistency: Cronbach’s alpha = NR | NR | NR |
| Ashley’s questionnaire | Ashley, M. J. et al (2007) / Canadá [32] | Smoking cessation | Developed from a literature review, consultation with pharmacists; Face validity | NR | High internal consistency: Cronbach’s alpha for various scales > 0.8 | NR | NR |
| Mohamed’s questionnaire | Mohamed, S. S. et al (2014) / Sudão [33] | Chronic diseases management | Developed from the literature; Content and face validity: panel of five pharmacists and pilot | NR | Internal consistency: Cronbach’s alpha = 0,7 | NR | NR |
|  |  |  |  |  |  |  |  |
| Tai’s instrument | Tai, B. et al (2016) / EUA [34] | Medication disposal | Developed according to the Theory of Planned Behavior (TPB); Content and face validity: pre-test | NR | Internal consistency: Cronbach’s alpha > 0,70 | NR | NR |
| Paluck’s questionnaire | Paluck, E.C. et al (1994) / Colúmbia Britânica- Canadá [35] | Health education | Content and Face Validity: tested with pharmacists | NR | Internal consistency: Cronbach’s alpha = 0,93 | NR | NR |
| Mohamed’s questionnaire | Mohamed, S. S.-E. et al (2013) / Sudão [36] | Health education and promotion services to the public | Developed from the literature; Content and face validity: panel of five pharmacists and pilot | NR | Internal consistency: Cronbach’s alpha = 0,7 | NR | NR |
| Shah’s questionnaire | Shah, B. et al (2011) / New York [37] | Medication Therapy  Management | Content and Face validity | NR | Internal consistency: Cronbach’s alpha for the attitude section = 0.75 | NR | NR |
| MMAM instrument | Witry, M. J. et al (2016) / EUA [39] | Medication monitoring | Qualitative interviews, in the item development phase; Content validity: two employees and pilot test | NR | Internal consistency: Cronbach’s alpha 0.819-0.811;  Factor analysis (promax rotation) <0.500 | Concorrent validity and predictive validity | NR |
| Perreault’s questionnaire | Perreault, M. M. et al (2012) / Canadá [40] | Clinical research | Developed by an interactive process between researchers; Face validity: pre-test | NR | Correlation coefficients with test-retest reliability assessment> 0.70, English version = 0.741, French version = 0.841 | NR | NR |
| Stewart’s questionnaire | Stewart, D. et al (2015 ) / Catar [41] | Research capacity | Developed based on literature, document and theoretical frameworks; Content and Face validity | NR | Internal consistency: Cronbach’s alpha> 0.9 in sections | NR | NR |
| Guirguis’s questionnaire | Guirguis, L. M. et al (2018) / Canadá [42] | Prescription | Developed from the literature and qualitative studies; Content and face validity | NR | Exploratory factorial analysis | NR | NR |
| Elkalmi’s questionnaire | Elkalmi, R. M. et al (2014) / Malásia [43] | Adverse Drug Reaction Reporting | Developed a literature review and based on a qualitative study; Content and face validity | NR | Internal consistency: Cronbach’s alpha = 0,871. | NR | NR |
| Taing’s questionnaire | Taing, M. et al (2016) / Austrália [44] | Oral healthcare | Developed based on previous studies and authors’ experience; face validity: tested with pharmacists | NR | Internal consistency: Cronbach’s alpha between 0,8 e 0,9 | NR | NR |
| Zardain’s  questionnaire | Zardain Tamargo, E. et al (2006) / Espanha [45] | Pharmacotherapeutic follow-up | NR | NR | Internal consistency: Cronbach’s alpha Factor 1 = 0.96; Factor 2 = 0.95; Factor 3 = 0.94; Factor 4 = 0.87. | NR | NR |

*NR- Not reported
